# Effects of brood size manipulation and common origin on phenotype and telomere length in nestling collared flycatchers

**DOI:** 10.1186/1472-6785-12-17

**Published:** 2012-08-18

**Authors:** Marie Voillemot, Kathryn Hine, Sandrine Zahn, François Criscuolo, Lars Gustafsson, Blandine Doligez, Pierre Bize

**Affiliations:** 1 Department of Ecology and Evolution, Biophore, University of Lausanne, CH-1015, Lausanne, Switzerland; 2 Département d’Ecologie, Physiologie et Ethologie, CNRS, Université de Strasbourg, IPHC, F-67087 Cedex 2, Strasbourg, France; 3 Department of Animal Ecology, Evolutionary Biology Centre, Uppsala University, SE-752 36, Uppsala, Sweden; 4 Department of Biometry and Evolutionary Biology, CNRS, Université de Lyon, Lyon; Université Lyon 1, F-69000, LBBE UMR 5558, Bâtiment Gregor Mendel, 43 boulevard du 11 november 1918, F-69622, Villeurbanne, France

## Abstract

**Background:**

Evidence is accumulating that telomere length is a good predictor of life expectancy, especially early in life, thus calling for determining the factors that affect telomere length at this stage. Here, we investigated the relative influence of early growth conditions and origin (genetics and early maternal effects) on telomere length of collared flycatchers (*Ficedula albicollis*) at fledging. We experimentally transferred hatchlings among brood triplets to create reduced, control (i.e. unchanged final nestling number) and enlarged broods.

**Results:**

Although our treatment significantly affected body mass at fledging, we found no evidence that increased sibling competition affected nestling tarsus length and telomere length. However, mixed models showed that brood triplets explained a significant part of the variance in body mass (18%) and telomere length (19%), but not tarsus length (13%), emphasizing that unmanipulated early environmental factors influenced telomere length. These models also revealed low, but significant, heritability of telomere length (*h*^2^ = 0.09). For comparison, the heritability of nestling body mass and tarsus length was 0.36 and 0.39, respectively, which was in the range of previously published estimates for those two traits in this species.

**Conclusion:**

Those findings in a wild bird population demonstrate that telomere length at the end of the growth period is weakly, but significantly, determined by genetic and/or maternal factors taking place before hatching. However, we found no evidence that the brood size manipulation experiment, and by extension the early growth conditions, influenced nestling telomere length. The weak heritability of telomere length suggests a close association with fitness in natural populations.

## Background

Understanding the process of ageing and the factors that influence individual’s lifespan is a major focus in evolutionary biology and biomedical sciences. A growing body of evidence suggests that genome integrity maintenance is essential to guarantee healthy organismal ageing, and that telomeres have an important role in this maintenance process [1]. Telomeres are highly conserved non-coding DNA repeat sequences that cap the end of linear eukaryotic chromosomes (typically repeats of (TTAGGG)_n_ in vertebrates and (TTAGG)_n_ in insects; [2,3]). In so doing, they prevent the end of chromosomes of being wrongly recognized as broken or damaged [4]. Because DNA double-stranded break activates DNA damage response pathways and induces cell cycle arrest and apoptosis [5,6], the presence of ‘healthy’ telomeres is essential to distinguish damaged chromosomes from healthy ones [5,6]. Interestingly, because DNA polymerase is unable to copy the very end of chromosomes during replication of normal somatic cells (the so-called ‘end replication problem’), telomeres are shortening at each cell division until they reach a critical size below which cell replicative senescence is triggered [4,7,8]. Although telomeres can be restored by the reverse transcriptase enzyme known as telomerase, this enzyme is mainly active in germinal and stem cells [4-6]. High telomerase activity in somatic cells has been linked to cellular proliferation and cancer [9], and down-regulation of telomerase in somatic tissues is thought to have evolved as a tumor suppressing mechanism [10]. Hence, it has been suggested that telomere dynamics has an important influence on organismal ageing [11,12], and that inter-individual variation in telomere length predicts, at least partially, inter-individual variation in life expectancy [13]. Accordingly, telomere length measured early in life or at adulthood has been found to predict subsequent survival, and in turn life expectancy, in organisms as diverse as humans [14-17], mice [18], lizards [19] and birds [20-24]. The relative contributions of genetic and environmental factors on the large inter-individual variation in telomere length among individuals of the same age remain elusive, and this advancement could help understanding the nature of the large inter-individual variation in life expectancy [13,25,26].

The study of human genetic disorder and of genetically modified organisms has provided abundant evidence of diverse genetic pathways involved in the regulation of telomere length (reviewed in [5,6,27]). Recent genome-wide association surveys in yeast [28] and humans [29,30] have also confirmed that telomere length is a polygenic trait. Yet, how much of the variation of telomere length in a population is transmitted to the next generation (i.e. heritable) has received little attention despite its importance in our understanding of the response to selection and evolvability of telomere length, and putatively life expectancy [31]. Presently, available heritability estimates of telomere length come almost exclusively from comparisons between twins or parent-offspring in humans, with reported heritability estimates ranging between 0.36 and 0.82 [16,29,32-35]. To our knowledge, only two studies so far have investigated heritability of telomere length in wild animals, showing significant heritability ranging between 0.52 and 1.23 in the sand lizard (*Lacerta agilis*; [19] and between 0.80 and 2.05 in the kakapo (*Strigops habroptilus*; [36]; T. Horn, pers. com.).

Besides genetic factors, there is increasing evidence that telomere length is influenced by environmental factors acting during development [37-40] and by adult lifestyle [41-43]. The effects of the environment on telomere length can be substantial (e.g. explaining up to 50% of the variance in telomere length [29]), and early life conditions are probably particularly important in shaping telomere length due to fast cell divisions during development [44-46]. Environmental factors leading to increased oxidative stress and DNA damage can further accelerate telomere erosion [47]. Early life conditions have been reported to profoundly influence later life, and thus telomeres have also been suggested as potential effectors linking early life conditions to later organismal ageing [25,48,49]. Accordingly, a recent longitudinal study on captive zebra finches (*Taeniopygia guttata*) has demonstrated that telomere length at 25 days of age was a strong predictor of realized lifespan [24]. How early growth conditions affect telomere length and subsequent survival remains poorly known [39] and warrants further studies, especially in natural populations where organisms are subjected to large environmental variations.

Here, we experimentally studied the relative importance of origin (i.e. genetics and/or early maternal effects) and early growth conditions on telomere length close to the end of the growth period in a natural population of an altricial bird species, the collared flycatcher (*Ficedula albicollis*) (see [50] for information on the study site and species). To do so, we performed a brood size manipulation experiment in the study population: broods hatched on the same day were matched in triplets, and part of the hatchlings were exchanged among broods of each triplet to create one brood reduced by two nestlings, one control brood (i.e. non-manipulated number of nestlings) and one brood enlarged by two nestlings within each triplet. We then investigated the effect of the brood size manipulation experiment on nestling body mass, tarsus length and telomere length close to fledging, and estimated the heritability of these three nestling phenotypic traits by comparing siblings reared in different nests (i.e. sib-sib design). We split the total variance of each nestling phenotypic trait using linear mixed models where we entered as explanatory variables the brood size manipulation treatment as one fixed factor and the nest of origin and the triplet as two random factors [51]. The ‘triplet’ factor controls for any difference in a group of nests (e.g. due to time in the season or spatial variation) and the ‘origin’ factor accounts for variation due to genetic and early maternal effects. Heritability (*h*^2^) was estimated by using the variance components given by the linear mixed model as: *h*^2^ = V_A_ / V_P_, where V_A_ represents the additive genetic variance, and V_P_ the total phenotypic variance (V_P_ = V_A_ + V_T_ + V_R,_ with V_A_ = additive genetic variance, i.e. associated to ‘origin’, V_T_ = environmental variance explained by ‘triplets’ and V_R_ = residual error). Telomere length was quantified using a real-time quantitative PCR developed to measure relative telomere length in birds [52].

## Results

**Figure 1:**
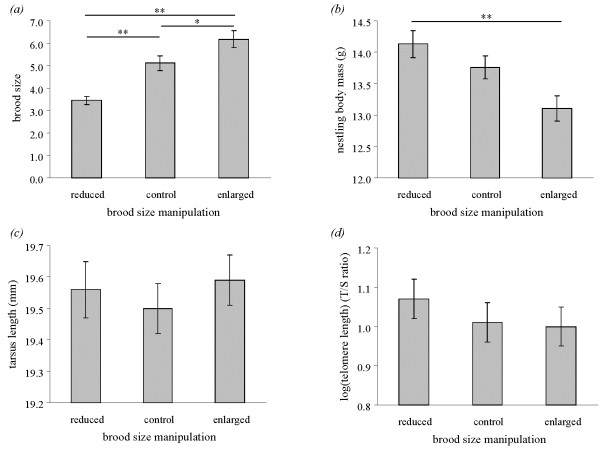
**Effect of the experiment on final brood size and nestling phenotype.** Mean ± s.e. (***a***) number of 12-day-old nestlings per experimental nest, (***b***) nestling body mass, (***c***) tarsus length, and (***d***) log-transformed relative telomere length. Significant differences among treatments are highlighted with lines; *: *p* < 0.05 ; **: *p* < 0.0001).

Before the brood size manipulation, there was no significant difference in the number of two-day-old nestlings between reduced (mean number of nestlings ± s.e. = 5.3 ± 1.6, number of broods *n* = 26), control (5.4 ± 1.6, *n* = 26) and enlarged broods (6.1 ± 1.0, *n* = 22; Kruskal-Wallis test: χ^2^ = 2.86, d.f. = 2, *p* = 0.24). Ten days after the manipulation, there were fewer nestlings in reduced compared to control broods and in control compared to enlarged broods (χ^2^ = 28.01, d.f. = 2, *p* < 0.001; Figure 1*a*), showing that the treatment was efficient, but nestlings were heavier in reduced compared to enlarged broods (Table 1; Figure 1*b*). Brood size manipulation had no significant effect on nestling tarsus length (Table 1; Figure 1*c*) and relative telomere length (Table 1; Figure 1*d*).

**Table 1:** Results of mixed-models analyzing nestling phenotypic variance in relation to brood size manipulation experiment (fixed effect), nest of origin and nest triplet (random effects; see text)

	**Body mass**				**Tarsus length**				**Relative telomere length**			
**Fixed effects**	**d.f.**	***F***	***p-*value**		**d.f.**	***F***	***p-*value**		**d.f.**	***F***	***p-*value**	
brood size manipulation	2,273.5	11.210	< 0.001		2,272.2	0.918	0.400		2,166.3	0.792	0.455	
**Random effect**	**var. comp.(s.e.)**		**LRT**	***p-*value**	**var. comp. (s.e.)**		**LRT**	***p-*value**	**var. comp. (s.e.)**		**LRT**	***p-*value**
nest of origin	0.747 (0.210)		23.113	< 0.001	0.148 (0.042)		21.994	< 0.001	0.014 (0.009)		2.159	0.038
nest triplet	0.374 (0.220)		2.141	0.039	0.047 (0.038)		0.870	0.187	0.027 (0.012)		4.460	0.003
error	0.951 (0.083)				0.190 (0.016)				0.116 (0.010)			

Significance of fixed effects were tested using Wald *F*-statistics and of random effects using log-likelihood ratio tests (LRT).

**Figure 2:**
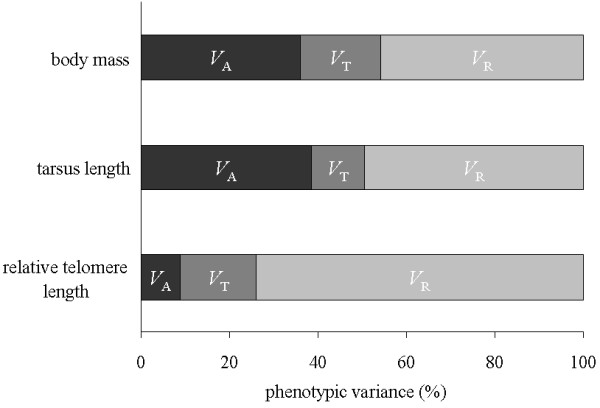
**Partitioning of nestling phenotypic variance.** Results of linear mixed models partitioning nestling phenotypic variance (*V*_P_) of body mass, tarsus length and relative telomere length into additive genetic variance (V_A_), environmental variance associated with brood triplets (*V*_T_) and residual error (*V*_R_), after controlling for the variance explained by the brood size manipulation experiment. $V_{P}=V_{A}=V_{T}=V_{R}+\text{and}\;h^{2}=V_{A}/V_{\mathrm{P.}}$

After controlling for the brood size manipulation, our linear mixed models showed significant additive genetic variance and/or maternal effects (i.e. here, effect of nest of origin) on nestling body mass, tarsus length and relative telomere length (Table 1). This translated into heritability estimates of *h*^2^ = 0.36 for body mass, *h*^2^ = 0.39 for tarsus length and of *h*^2^ = 0.09 for relative telomere length (Figure 2). There was a significant effect of environmental conditions other than growth conditions (i.e. here effect of triplet) on relative telomere length and body mass but not on tarsus length (Table 1). Because one important source of non-manipulated early environmental conditions associated to the effect of triplet is the hatching date, we computed two additional mixed models where hatching date was entered as a fixed covariable to investigate its influence on nestling body mass and telomere length and, in turn, heritability estimates. Hatching date was significantly negatively related to nestling body mass, as could be predicted (*p* = 0.02), but not to telomere length (*p* = 0.47). As a consequence, including this covariable slightly increased the heritability estimate of body mass (*h*^2^ = 0.38 instead of 0.36) but did not change the estimate for relative telomere length (*h*^2^ = 0.09). We did not test it for tarsus length because the effect of triplet was not significant for this variable.

Nestling sex had no significant influence on 12-day-old nestling body mass (*p* = 0.09), tarsus length (*p* = 0.50) or relative telomere length (*p* = 0.82). Thus, the effect of nestling sex was dropped from the final models.

## Discussion

Variation in telomere length has been demonstrated to predict subsequent survival in numerous organisms [14-23]. Fascinatingly, a recent study in a captive population of zebra finches where telomere length was measured at various time points throughout the life of each individual showed that telomere length in early life (i.e. 25 days after hatching, which coincides with the end the post-fledging parental care) was the strongest predictor of realized lifespan [24]. Furthermore, although individuals with long telomeres early in life maintained longer telomeres throughout their life compared to individuals with short telomere early in life, telomere length early in life is a much stronger predictor of realized lifespan when compared to telomere length in adulthood [24]. These findings emphasize the importance of understanding the factors that determine telomere length early in life. Here, we report results from a brood size manipulation experiment testing the relative importance of early growth conditions and genetics and early maternal effects on telomere length measured close to the end of the growth period in nestling collared flycatchers. In agreement with previous studies in this and other passerine species [51,53,54], offspring raised in enlarged broods, and thus facing increased sibling competition and lower access to food, were in significantly poorer condition compared to offspring raised in reduced broods, as reflected by lower body mass. However, we found no evidence that the brood size manipulation significantly influenced offspring structural body size, as measured by tarsus length, and telomere length. Body mass is known to quickly respond to short time environmental changes; skeletal traits are less sensitive to environmental variation [51,55]. Hence, a significant effect of treatment on fledgling body mass, but not tarsus length, is not surprising*.* Yet, the lack of significant effect of treatment on telomere length is more surprising, and it emphasizes that telomere length at the end of the growth period might not accurately reflect the past developmental conditions after hatching in this bird species.

The lack of difference in telomere length between experimental treatments however needs to be interpreted with caution for the three following reasons. First, telomere length was measured using red blood cells present in the blood sample; red blood cells have a turnover of 30 to 40 days in birds [56]. Thus, we cannot exclude that the effect of early growth conditions on telomere length in red blood cells will become apparent only 15 to 25 days after fledging, which takes place around 16 days of age in the collared flycatcher. Second, our mixed models revealed that part of the variation in offspring telomere length and body mass, but not tarsus length, was significantly explained by the effect of nest triplet. Because broods matched in triplets had hatched on the same day and were in most cases located in the same forest plot, this random term is controlling, among other things, for seasonal and spatial effects on telomere length. Hence, the significant nest triplet effect on offspring telomere length suggests that some non-manipulated environmental factors have influenced telomere length, and thereby that telomere length is sensitive to environmental conditions. Here, note that additional analyses pointed out that hatching date was not affecting telomere length, and thus important early environmental variables remain to be identified. Finally, 2011 was a very good year for breeding and the development of nestling collared flycatchers in our study site, with a nest failure probability (i.e. no fledglings produced) from 20 to 50% lower than in the previous years (B. Doligez, unpublished data). Thus, replicating this brood size manipulation in years with contrasting environmental conditions is needed to investigate the importance of cohort and treatment effects on variation in offspring telomere length at fledging.

Our mixed models showed a significant effect of the nest of origin on offspring body mass, tarsus length and telomere length, which translated into significant heritability estimates for these three traits. The low but nonetheless significant estimate of heritability of telomere length (*h*^2^ = 0.09) in fledgling collared flycatchers contrasts with previously published heritability estimates, which were notably higher [16,19,29,32-36]. At least three reasons can be evocated to explain discrepancies in telomere length heritability across studies. First, most heritability estimates reported in previous studies are based on telomere restricted fragment (TRF) measurements rather than quantitative PCR measurements. Although the two methods have been demonstrated to produce comparable telomere measurements in humans [57], mice [58] and birds [52], we cannot exclude that lower heritability estimates in flycatchers are rooted in the methodological approach to quantify telomere length [59]. Both TRF and qPCR measurements present methodological difficulties to accurately estimate telomere length [60], and systematic variation in measurement errors of telomere length between the two methods could hamper comparisons of heritability estimates among studies. Second, maternal effects can inflate sibling resemblances [42], calling for caution when interpreting heritability estimates based on sib-sib comparison (e.g. [32-34]) or on parent-offspring regression (e.g. [19,36]). However, the heritability estimates obtained here for body mass (0.36) and tarsus length (0.39) are consistent with previous estimates obtained in the same study population using a mixed model [60]. Therefore, this gives confidence in the low heritability estimate of telomere length found in the present study. Finally, telomere length and its heritability level frequently differ between the sexes [16,19,34,36], and it has been proposed that telomere length is primarily inherited from the heterogametic sex [36], namely fathers in humans [16] and in some reptiles as the sand lizard [19] and mothers in birds [36]. Here, we found no difference in telomere length between the sexes in nestling collared flycatchers, but computing sex-specific heritability estimates would be needed to assess whether our estimate could be affected by this inheritance mechanism. Unfortunately, the models did not converge when computing sex-specific estimates because of a limited number of opposite-sex siblings reared in different nests (n = 28 families for sister-sister comparisons and 19 for brother-brother comparison).

Additional studies in the wild are required for assessing the relative importance of early growth conditions and genetics and maternal effects on telomere length measured at various ages to better understand the factors shaping the variation in telomere length in natural populations and for testing the links between telomere length in early life and later survival. Strong natural selection on a phenotypic trait can quickly deplete its additive genetic variation and, in turn, its heritability [61]. The lower heritability of telomere length compared to body mass and tarsus length may supports the hypothesis that telomere length is under stronger selection, and in turn is more closely associated with fitness in this natural population. Assessing the shape and strength of natural selection on telomere length is needed to test this hypothesis and get insights on the evolutionary potential of telomere length in the wild.

## Conclusion

This study shows that telomere length of nestlings close to fledging was weakly, but nonetheless significantly, influenced by genetics and/or maternal effects taking place before hatching in a natural populations of birds. Furthermore, although the brood size manipulation experiment was efficient at altering nestling body mass, there was no evidence that this experimental manipulation of nestling early growth conditions affected the length of their telomeres.

## Methods

### Study species and population monitoring

The collared flycatcher is a small, cavity-breeding, migratory bird that reproduces in deciduous and mixed forests. Most males are monogamous and females lay one clutch per year of 5 to 7 eggs (rarely 4 or 8 eggs) that they incubate alone during 13–14 days. Both parents feed the young mainly with caterpillars; young leave the nest between day 15 and 18. Data were collected in spring 2011 in a population of collared flycatchers breeding on the southern part of the Island of Gotland, Southern Baltic (57°10’N, 18°20’E), monitored since 1980, where nest boxes have been provided to the birds in spatially discrete forest plots and are readily accepted by the birds. Each year, nest boxes are monitored throughout the season to assess occupancy, egg laying date, clutch size and number of hatchlings and fledglings. All nestlings are ringed before fledging; mothers are trapped, identified and measured at the incubation stage and social fathers while provisioning the brood. Additional information about the study area and the breeding ecology of the species can be found in [50].

### Brood size manipulation experiment

On the second day after hatching, broods hatched on the same day were matched in triplets, and we experimentally manipulated brood sizes by transferring two nestlings from nest A, the reduced brood size nest, into nest B, the control nest, and two nestlings from nest B into nest C, the enlarged brood size nest. By comparing siblings raised in different nests, this design allowed to separate genetic and early maternal effects occurring before hatching from environmental effects occurring after hatching. Nestlings were individually identified at cross-fostering using unique nail clipping combinations, and correspondence with ring number was done at ringing, on day 8 after hatching. Body measurements were taken at day 12, during the last visit to the nest to avoid premature fledging. Nestlings were weighed to the nearest 0.1 g, their tarsus length measured to the nearest 0.1 mm, and a blood sample was collected from the wing for molecular sexing and measurement of telomere length. Nestlings were exchanged within 36 triplets in total, and for logistic reasons (i.e. space constraint to run all the samples on one 384-well qPCR plate; see below) we restricted our analyses to 30 triplicates, choosing preferentially triplets with available data on 12-day-old nestlings in the three nests of the triplet.

### Genomic DNA extraction

Blood samples were collected in EDTA-coated tubes, stored on cold packs in the field before being centrifuged in the same evening to separate plasma from red blood cells (RBC). Plasma and RBC were stored at −80°C until later analyses in the laboratory. Genomic DNA was extracted from RBC samples using DNeasy Blood & Tissue Kit (Qiagen©) and by following the manufacturer protocol. DNA quantification was performed using ND-1000-Spectrophotometer (NanoDrop Technologies).

### Molecular sexing

Nestlings were sexed based on two CHD genes on the avian sex chromosomes and using the primers P2550-F (5^′^-GTTACTGATTCG-3^′^) and P2757-R (5^′^-AATTCCCCTTTTATT-3^′^) [62]. For a final volume of 10 μl, we used 0.8 μl of each primer (0.1 μg/μl), 0.8 μl of dNTP (2.5 mM), 0.8 μl of MgCl2 (25 mM), 2 μl of GoTaq® Green buffer, 2.7 μl of H_2_O, and 2 μl of DNA sample. The thermal profile comprised an initial denaturing step of 94°C for 2 min, followed by 30 cycles of 45 s at 94°C, 1 min at 49°C, 1 min at 72°C, and a final extension step of 5 min at 72°C that was added after the last cycle. PCR products were separated in a 2% agarose-gel at 100 volts for 30 min and visualized by ethidium bromide staining. In birds, male is the homogametic sex, and thus a single Z-CHD-band indicates a male while the presence of a second W-CHD indicates a female.

### Telomere length measurements

Telomere length was quantified using a real-time quantitative PCR developed to measure relative telomere length in humans [57] and later validated in birds [52]. This technique estimates relative telomere length by determining the ratio (T/S) of telomere repeat copy number (T) to gene with a non-variable copy number (S) in focal samples. The non-variable copy number gene needs to have the same copy number among individuals in the population and within individuals over time. Here, we used 18S as a non-variable copy number gene. Forward and reverse telomere primers were 5^′^-CGGTTTG TTTGGGTTTGGGTTTGGGTTTGGGTTTGGGTT-3^′^ (Tel-1b) and 5^′^-GGCTTGCCTTACCCTTACCCTTACCCTTACCCTTACCCT-3^′^ (Tel-2b), respectively, and forward and reverse 18S primers were 5^′^-GAGGTGAAATTCTTGGACCGG-3^′^ and 5^′^-CGAACCTCCGACTTTCGTTCT-3^′^. Both primers were used at a concentration of 100 μM. qPCR primers also amplify non-telomeres (TTAGGG)_n_ sequences, such as interstitial sequences which are common in birds. However, interstitial repeats do not vary with age, and show little variation among individuals of the same species, so this should not be a limitation in the present study [63].

For the quantitative PCR assay, we used 1 ng of DNA per reaction, for a total volume of 10 μl (8 μl of master mix + 2 μl of DNA). The master mix contained 0.015 μl of each primer, 2.97 μl of water and 5 μl of Applied Biosystems® SYBR® Green PCR master mix (ref. 4309155; Life Technologies) per reaction. In order to improve the reaction, with the telomere reaction we added 0.09 μl of betaïne to the master mix (and subtracted this volume from the initial water volume). PCR conditions for telomere were 10 min at 95°C, followed by 40 cycles of 15 s at 95°C, 34 s at 60°C and 30 s at 72°C; and for 18S 2 min at 50°C, 10 min at 95°C, followed by 40 cycles of 15 s at 95°C and 1 min at 60^°^C. Both reactions ended with a dissociation program of 15 s at 95°C, 1 min at 60°C and 15 s at 95°C. PCR plates of 384 wells were loaded with a TECAN robot, thereby avoiding pipetting error and improving consistency and repeatability among plates. qPCR measurements were performed using an Applied Biosystems 7900 HT Fast Real-Time PCR System. Telomere and 18S amplifications were carried out on different 384-well plates, each one containing 369 samples, one serial dilution run in duplicates (two fold-dilution from 8 ng down to 0.125 ng of DNA per well) and one negative control. Each plate was replicated twice to obtain two telomere and 18S measurements for each sample. Serial dilutions were used to set up the threshold C_*t*_ value and to produce a standard curve allowing testing for the efficiency and goodness-of-fit of each PCR reaction. Mean amplification efficiencies and *r*^2^ of the qPCR runs were, were respectively, 97.2% and 0.986 for 18S and 95.1% and 0.965 for telomeres, which are in the ranges recommended by guidelines for qPCR experiments [64]. Thirteen samples showed inconsistent *C*_*t*_ values between the runs and were therefore excluded from the analyses. Mean ± s.e. intra-individual variation was 0.33% ± 0.01% for the 18S assays and 0.63% ± 0.02% for the telomere assays. Relative T/S ratios were calculated separately for each replicate using the following formula: ${\left(1+E_{\text{tel}}\right)}^{\Delta C\text{t tel}}/{\left(1+E_{18S}\right)}^{\Delta C\text{t 18S}}$, where E_tel_ represents the telomere plate efficiency, E_18S_ the 18S plate efficiency, and $\Delta C_{t}=C_{t}^{\mathrm{golden}}-C_{t}^{\mathrm{unknown}}$, where the golden sample is a sample chosen as a point of reference for the comparison of other samples (see introduction to quantitative PCR: methods and application guide by Stratagene 2007). Then, for each individual we computed mean relative T/S ratios over the two replicates.

### Statistical analyses

All analyses were performed using the library *asreml* in R.2.13.2 (CRAN, 2011). Relative telomere length measurements were log-transformed before analyses to homogenize the variance among treatments. Final molecular and statistical analyses were performed on 359 nestlings from 74 broods, with 90, 132 and 137 nestlings from, respectively, 26, 26 and 22 reduced, control and enlarged broods.

### Ethical note

The brood size manipulation experiment and sample collection were conducted under a licence from the Swedish National Board for Laboratory Animals, and bird catching and manipulating under a ringing licence from the Bird Ringing Centre of the Swedish Museum of Natural History (Stockholm, Sweden).

## Authors’ contributions

PB and BD conceived of the study. MV carried out the field work with the support of BD and LG, the molecular analyses with the support of KH, SZ, FC and PB, and the statistical analyses together with PB. MV, PB and BD wrote the paper. All authors read and approved the final manuscript.

## Author details

^1^Department of Ecology and Evolution, Biophore, University of Lausanne, CH-1015, Lausanne, Switzerland. ^2^Département d’Ecologie, Physiologie et Ethologie, CNRS, Université de Strasbourg, IPHC, F-67087 Cedex 2, Strasbourg, France. ^3^Department of Animal Ecology, Evolutionary Biology Centre, Uppsala University, SE-752 36, Uppsala, Sweden. ^4^Department of Biometry and Evolutionary Biology, CNRS, Université de Lyon, Lyon; Université Lyon 1, F-69000, LBBE UMR 5558, Bâtiment Gregor Mendel, 43 boulevard du 11 november 1918, F-69622, Villeurbanne, France.
